# Tumor in Transit: Intracardiac Leiomyomatosis

**DOI:** 10.7759/cureus.43764

**Published:** 2023-08-19

**Authors:** Abey S Abraham, Teuta Marsic, Gyan Das, Anand Mehta

**Affiliations:** 1 Cardiothoracic Anesthesiology, Cleveland Clinic Foundation, Cleveland, USA

**Keywords:** intracardiac leiomyomatosis, tricuspid valve mass, smooth muscle tumour, intravenous leiomyomatosis, leiomyoma

## Abstract

Intravenous leiomyoma is a rare condition that occurs when there is a vascular invasion of a pre-existing uterine leiomyoma. The tumor can metastasize to structures such as the heart and lungs. We discuss a case of metastasis to the heart resulting in severe tricuspid regurgitation.

Surgical intervention is the primary modality; usually a staged approach involving cardiac surgery along with abdominal and/or pelvic surgery. We want to highlight the importance of fully investigating right-sided cardiac masses. While there are common etiologies for these masses, one must maintain a high degree of suspicion for an intravenous leiomyoma, especially if a female has certain risk factors such as a prior history of fibroids or a hysterectomy. We also stress the importance of a multi-disciplinary team approach when providing care to these patients, along with reviewing all modalities of imaging.

## Introduction

Intracardiac leiomyomatosis (ICLM) occurs when there is an intracardiac extension of intravenous leiomyomatosis (IVL). There have been less than 100 reported cases of ICLM in the literature [1]. Given the rare nature of this disease, prompt diagnosis and surgical intervention are crucial. Intracardiac leiomyomatosis is one of several differentials for right-sided cardiac masses, hence the challenge in correctly identifying the etiology of these masses. We present a case highlighting an unusual presentation of ICLM, along with the necessary multi-disciplinary teamwork needed to ensure the best possible outcome.

## Case presentation

A 42-year-old female, American Society of Anesthesiologists (ASA) grade 3, with a medical history of uterine fibroids and pulmonary embolism, presented to our institution with persistent abdominal pain and nausea. She had undergone a total abdominal hysterectomy (TAH) and left oophorectomy two years prior. During her admission, CT and MRI imaging revealed new filling defects within the left internal iliac vein, concerning a thrombus measuring 3.3×4.6cm. As part of her preoperative work-up and transthoracic echocardiography (TTE) revealed normal biventricular function, severe tricuspid regurgitation, and a new highly mobile echogenic lesion on the tricuspid valve. A laparotomy with excision of the mass from the left iliac vein was planned, followed by open heart surgery (OHS) involving tricuspid valve mass resection and repair.

**Figure 1:**
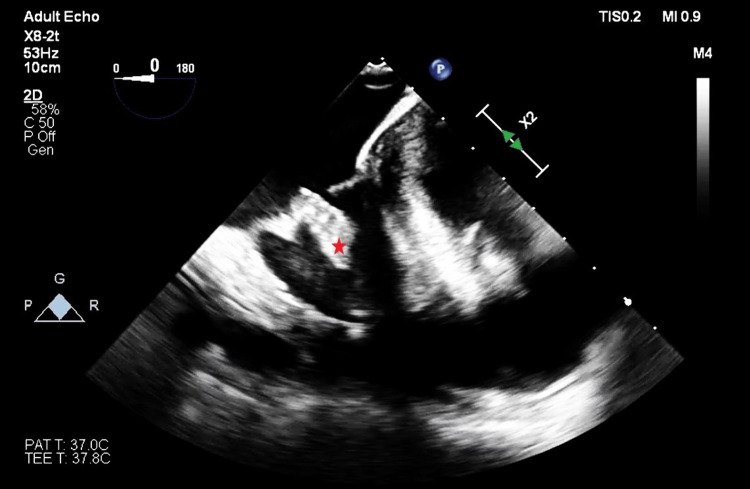
The TEE probe advanced from the mid-esophageal four-chamber view, depicting tricuspid valve mass (red asterisk) likely adhering to the posterior leaflet. TEE:  transesophageal echocardiography

**Figure 2:**
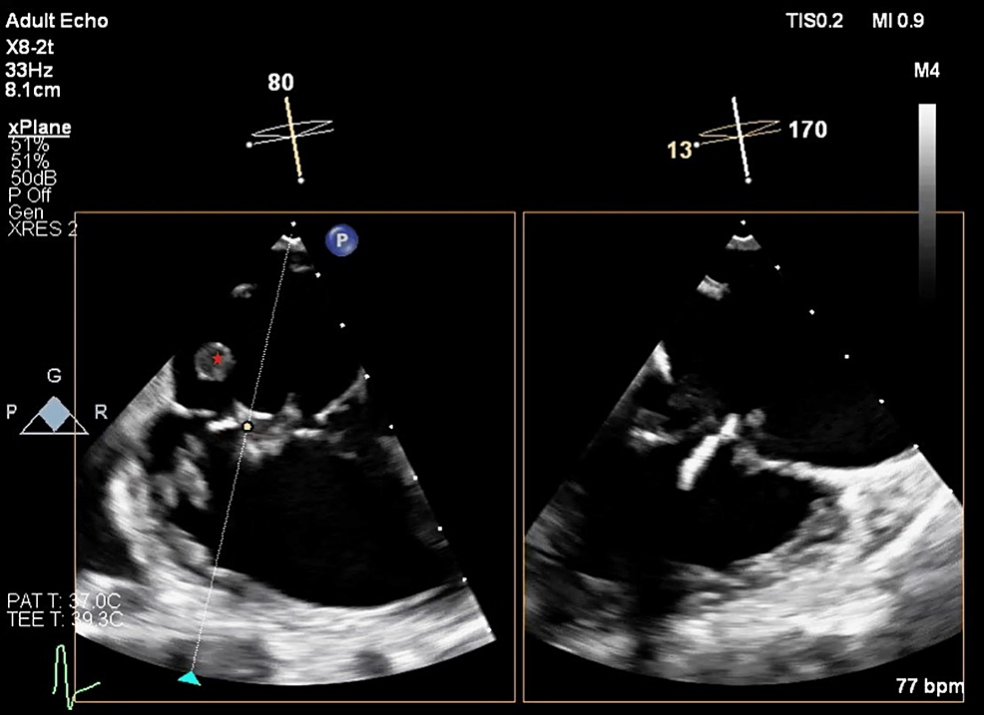
Mid-esophageal modified bicaval view (TEE) using multi-plane imaging (X-plane) showing an echogenic lesion (red asterisk) arising from the sub-valvular apparatus of the tricuspid valve

During her thrombectomy operation, intraoperative transesophageal echocardiography (TEE) was done for further evaluation of the echogenic lesions previously identified, along with monitoring for potential embolization. Although TEE revealed no change in systolic ventricular function or the severity of tricuspid regurgitation (TR), the tricuspid valve mass appeared larger and highly mobile, with concerns about embolization (Figures 1-2).

**Figure 3:**
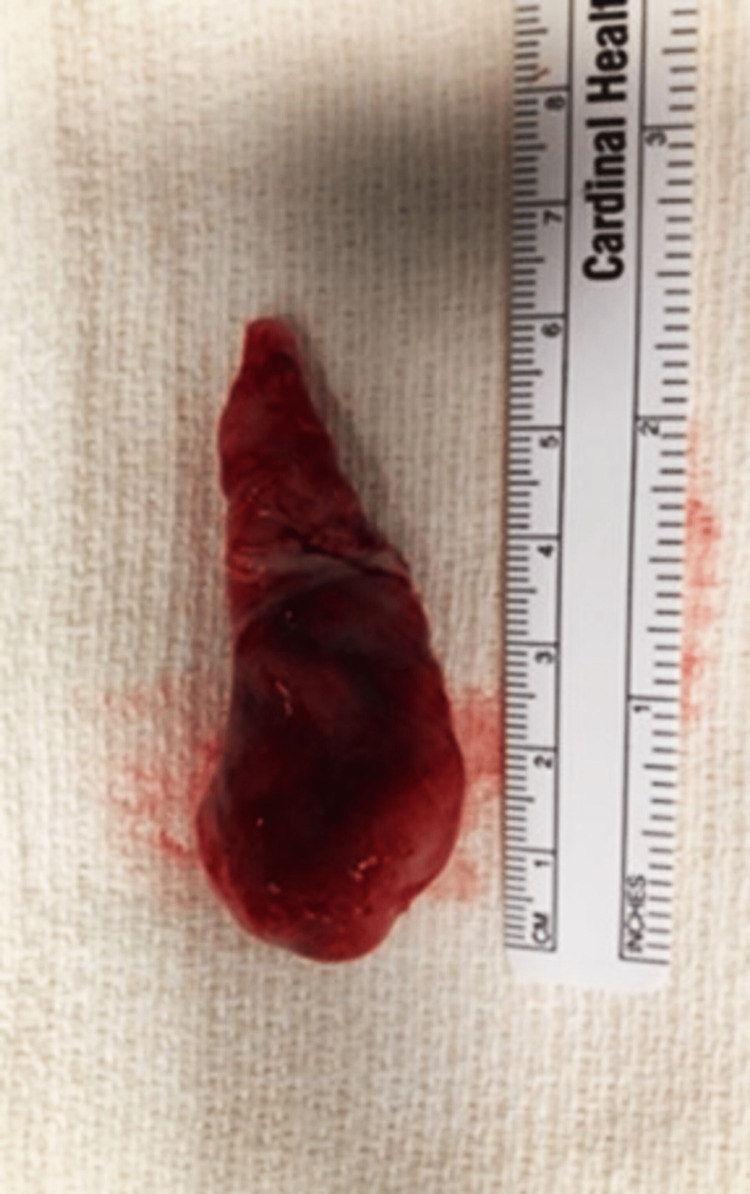
A 6-cm iliac vein mass that is solid and homogenous without hemorrhage or necrosis was resected.

**Figure 4:**
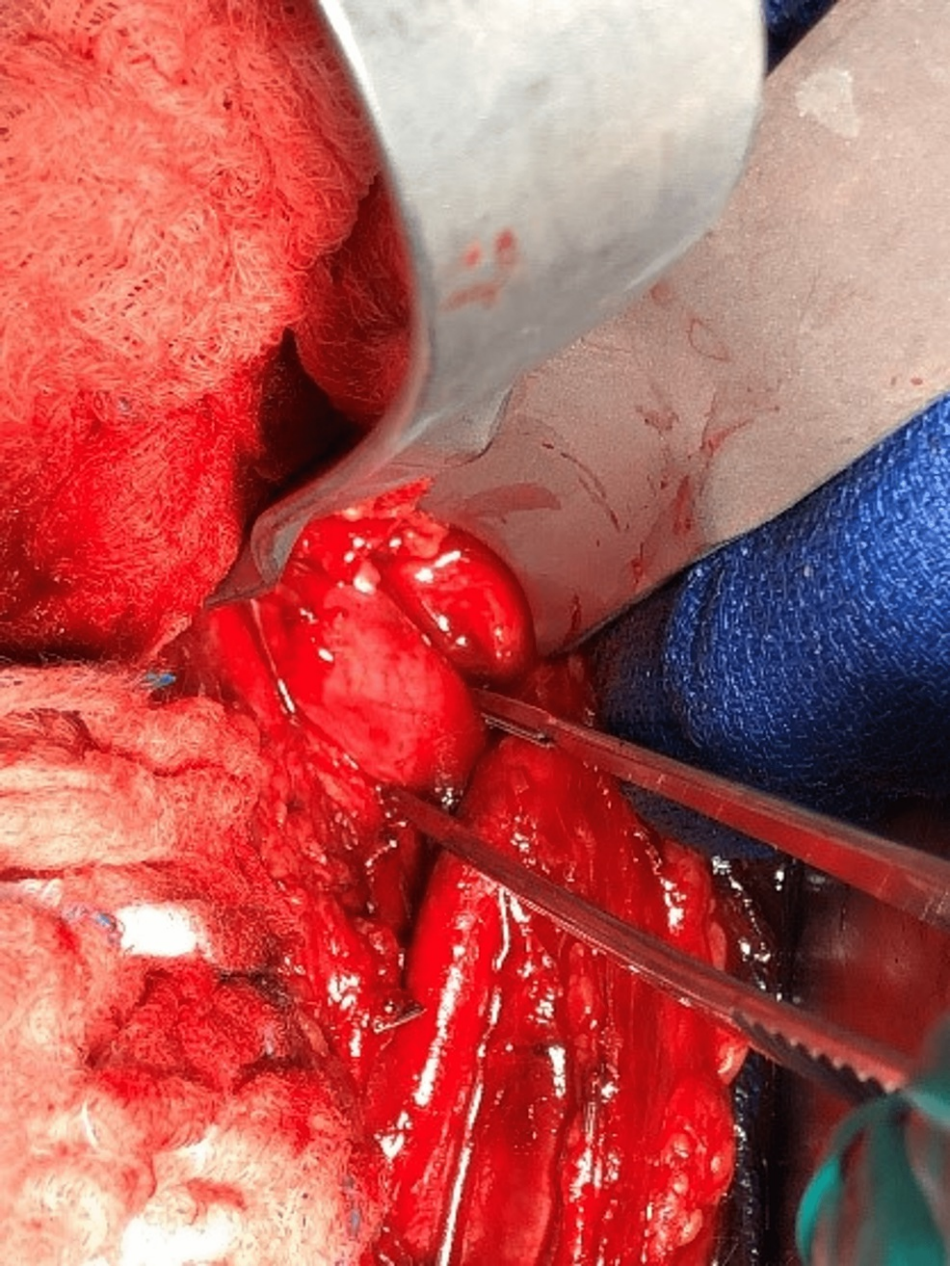
The application of a hemostat clamp holding the iliac vein mass

Thrombectomy was successful, with a 6 cm solid homogenous mass removed and sent to pathology (Figures 3-4).

A biopsy of this mass was consistent with intravenous leiomyomatosis (IVL).

Open heart surgery proceeded a month later, with pre-induction large-bore intravenous lines and a brachial arterial line being placed. The induction of anesthesia was uneventful. We obtained central venous access after the placement of the TEE probe. This helped guide the placement of the central line and prevented accidental embolization of the tricuspid valve mass with the wire or catheter. For similar reasons, we avoided the placement of a pulmonary artery catheter. Two masses were removed from within the subvalvular apparatus of the tricuspid valve, involving both anterior and posterior leaflets. Ultimately, a tricuspid valve repair was performed with an Edwards MC3 Size #30 annuloplasty ring. Cardiopulmonary bypass was weaned successfully without the need for inotropic support, and TEE revealed successful mass excision and trivial tricuspid regurgitation. At the end of the procedure, we performed bilateral pecto-intercostal fascial plane blocks for postoperative analgesia. She was transferred to a stepdown unit on postoperative day one and discharged home on postoperative day three.

**Figure 5:**
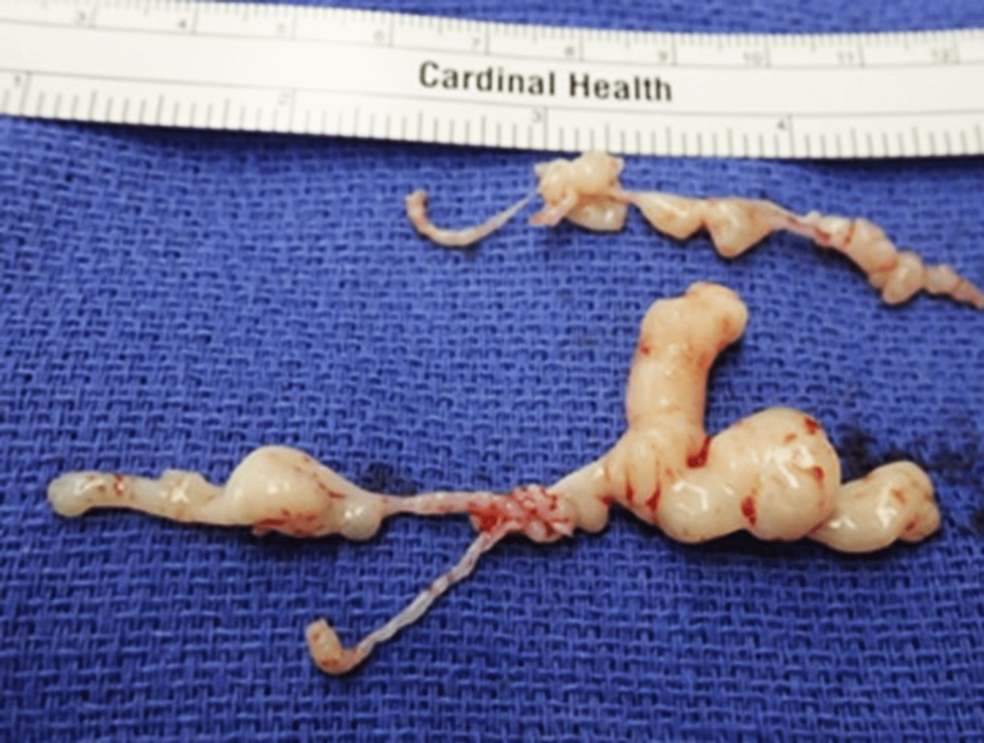
Two masses, measuring 1.0 x 0.4 x 0.1 cm and 5.0 x 2.5 x 1.0 cm, were extracted from the tricuspid valve.

The tricuspid valve masses were sent for surgical pathology. One mass measured 1.0 x 0.4 x 0.1 cm and the other 5.0 x 2.5 x 1.0 cm (Figure 5).

The findings revealed benign smooth muscle proliferation, along with bland oval to elongated nuclei, fine chromatin, and small nucleoli. No nuclear atypia or mitoses were seen. The lesional cells were diffusely positive for desmin and actin and focally positive for caldesmon, CD10, and CD34, which was consistent with a diagnosis of intravenous leiomyomatosis.

At the time of this report, 14 months from OHS, the patient appears to have developed lung nodules of unknown etiology.

## Discussion

Smooth muscle cardiac tumors are rare; however, when present, the etiology is typically a benign metastasizing leiomyoma, a primary cardiac smooth muscle tumor (leiomyoma), or an intracardiac extension of a remote smooth muscle tumor. Our patient had IVL, which is part of a sub-group of leiomyomas that extend beyond the uterus [2,3]. Intravenous leiomyomatosis typically affects middle-aged women, with a median age of 44 [4], with risk factors including prior hysterectomy, myomectomy, and a prior IVL diagnosis [2].

There is no definitive explanation for the pathophysiology of IVL; however, Steinmetz et al. [5] postulate that vascular invasion via a pre-existing uterine leiomyoma is possible. Furthermore, vascular spread into the inferior vena cava (IVC) is usually via the iliac veins or ovarian veins [2]. Our patient had a TAH two years prior, and despite this, there was evidence of IVL in the left iliac vein mass. One possible explanation is micrometastasis which was not detected earlier.

Given the rarity of IVL, there may be a delay in diagnosis or a chance of misdiagnosis, for example, attributing a right-sided cardiac mass to a right atrial myxoma. Other differentials worth considering include an extension of a tumor thrombus (TT) from a renal cell carcinoma, Budd-Chiari syndrome, propagation of a deep vein thrombus, or endometrial stromal sarcoma [6]. With these differentials, one can appreciate the difficulty in discerning between a thrombus and a tumor in transit.

We would like to highlight a tumor thrombus from renal cell carcinoma (RCC), which has been known to invade the right side of the heart. As per the Mayo Clinic Classification [7], stage 4 refers to TT extending above the diaphragm and into the right atrium. This typically warrants the use of circulatory arrest and cardiopulmonary bypass to successfully remove the tumor. With respect to histological appearance, ICLM is described as having oval-shaped nuclei along with positive desmin and actin expression. Furthermore, the tumor itself is not adherent to the vascular endothelium, possibly attributed to turbulent venous blood flow [8]. With TT from an RCC, there may be adherence with the vascular endothelium, along with possible invasion through the inferior vena cava wall [7]. Given the overlap in management and presentation of TT versus ICLM, a multi-disciplinary approach is crucial to ensuring the best possible outcome. For our patient, the teams involved included general surgery, vascular surgery, hematology and oncology, gynecology, cardiothoracic surgery, and cardiothoracic anesthesia.

Some of the feared complications associated with IVL include right ventricular outflow obstruction, recurrent pulmonary embolism, and cardiac arrest [4]. Surgical resection of the cardiac mass along with the primary source is considered the mainstay of treatment and is deemed to be curative. Surgical interventions for the primary source include total hysterectomy and bilateral salpingo-oophorectomy [8]. Furthermore, these patients must avoid exogenous estrogen-containing therapy, as estrogen has been shown to induce the growth of intravenous leiomyomas [9].

## Conclusions

In summary, when dealing with patients with right-sided atrial masses, it is essential to maintain a high degree of suspicion for ICLM, especially if there is a known history of fibroids and prior hysterectomy. We also discuss the importance of multi-disciplinary teamwork, along with using the relevant imaging modalities to help consolidate the correct diagnosis.
